# Dr. S. B. Browu Dead—Was the Oldest Practicing Dentist in Fort Wayne

**Published:** 1897-04

**Authors:** 


### BIOGRAPHICAL.

### Dr. S. B. Brown Dead—Was the Oldest Practicing Dentist in Fort Wayne.

Seneca Buel Brown, of Ft. Wayne, died January 5th, 1897. He was born August 11, 1834, at Marlboro, Windham County, Vermont. His father John Brown, was born in the city of Rochester, County Kent, England, August 28, 1787, and came to America and located at Rutland, Vt., in 1820. He married a daughter of Archelaus Dean, a native of Massachusetts, of Puritan ancestry, who served through the Revolutionary War and died in Vermont in 1846. John Brown, who was a member of the Church of England, and in politics a Free Soil Democrat, died at Westminster, Vermont, March 3, 1851. To the latter town the family removed when Dr. Brown was four years of age. Here, farm-life occupied him until 1852. In the meantime, the district school and three terms at Westminster Seminary completed his education. November 1, 1852, he entered the office of Oramel R. Post, D. D. S., at Battleboro, Vermont, as a student of dentistry, and on January 1, 1854, began practice at Ticonderoga, New York on a circuit including Ticonderoga, Westport, Essex, Elizabethtown and Schroon Lake, in Essex, and Chestertown, in Warren County. He came west and located at Piqua, Ohio, October 23, 1855, and there, on June 9, 1864, he was married to Nannie Louise eldest daughter of the Hon. Stephen Johnson, of that city. July 6, 1874, nine years after their removal to Fort Wayne, Mrs. Brown died leaving an only child, Katie, then seven years of age. February 14, 1888, he was married to Minnie Russell Graves, oldest daughter of Charles E. Graves, of Fort Wayne, who survives him. Dr. Brown came to Fort Wayne May 3, 1865, when the population of the city was 15,000, and at the time of his illness was the only resident dentist who was practicing in his own name in Fort Wayne in 1865. Dr. Brown received the degree Doctor of Dental Surgery from the Pennsylvania Dental College, March, 1870; was elected Secretary of the Indiana Dental Association June 29,

1869; received the degree of Doctor of Dental Surgery from the Ohio College of Dental Surgery March 1, 1871; was elected President of the Mississippi Valley Dental Association, the oldest in the world now in existence, March 6, 1874; was elected a member of the Board of Indiana Dental Examiners June 29, 1880, holding the office seven years; was elected President of the Indiana State Dental Association July 1, 1885, and a member of the Board of Trustees of the Indiana Dental College March 3, 1886; received the honorary degree of Doctor of Medicine from the Fort Wayne College of Medicine March 6, 1888, and March 6, 1889, was elected President of the Indiana Dental College. For over twenty-five years he has been a member of both the American and Indiana State Dental Associations. Dr. Brown's eminent skill in his profession, and his high personal character were the commanding influences which won for him an extensive clientage and a satisfactory mead of success. In politics Dr. Brown was a staunch believer in the principles of the Republican party. He was the first President of the Fort Wayne Colony of the American Sons of the Revolution and took an active interest in that society.

Three years ago Dr. Brown's health began to fail. He had been rugged and strong throughout his lifetime, and the advance of illness was soon detected in his vigorous frame. He was able to be about, however, and attended to his practice with regularity. Early in the present winter he failed perceptibly, and was soon commpelled to relinquish the active duties of life.

On Christmas Eve he was about the house, but the following day he was unable to leave his bed, and from that day his illness was recognized as of a character which aroused grave fears. At times his life seemed to hang in the balance, but his remarkable vitality would come to the rescue and there would be a period of comparative ease, and so it continued until the last, when death claimed him.

In the passing away of Dr. Brown Fort Wayne loses an honorable, progressive and respected citizen; the medical and dental professions lose a member who was an ornament and an honor to them, and his sorrowing widow is left to mourn the loss of one whose every thought was for the gladdening of their home-life. As a citizen no man can lay claim to a higher mead of praise than he. Quiet and unassuming in his habits, deeply versed in medical lore and current literature, his mind was a store-house of knowledge, valuable to himself and to those with whom he came in contact in his daily life, either as a practitioner or as a citizen. Affectionate and kind in temperament, he was loved by those around him, while his strict integrity and uncompromising honesty made him honored among men in all walks of life. To the members of his profession he was an adviser and a friend. His feeling toward his professional brethren is illustrated in a small sketch of his life written by himself some half a dozen years ago. He says:

"My interest in and attachment for my professional brethren has made my association with them one of the greatest enjoyments of my life. Without this stimulus and encouragement my practice would have been comparatively a blank."

Dr. Brown was deservedly proud of his ancestry, and the fact that his forefathers shed their blood for American liberty was a circumstance which occasioned him a degree of pride altogether pardonable and proper. He was one of the organizers of the Fort Wayne Colony of the Sons of the American Revolution, composed of the descendants of revolutionary patriots, and was the first president of that organization.

In the death of Dr. Brown, Fort Wayne will mourn the loss of one who, through a long residence, has ever retained the highest respect of the people of all classes, and a man against whom the breath of calumny has never been breathed. His life was stainless as his end was peaceful. A firm believer in the principles of republicanism, his political preferences were never obtrusive, and his course was that of a true American citizen, blameless, sincere and withal friendly.

He was a regular attendant at the Episcopal Church, and while his religion was never obtrusive his private and public life was molded on the principles of the Christian religion, and in accordance with the teachings of his youth.

